# Fluorescent-dependent comparative *C_t_* method for qPCR gene expression analysis in IVF clinical pre-implantation embryonic testing

**DOI:** 10.1093/biomethods/bpab001

**Published:** 2021-01-18

**Authors:** Arnav Lal, William E Roudebush, Monica Mainigi, Renee J Chosed

**Affiliations:** 1 School of Arts and Sciences, University of Pennsylvania, Philadelphia, PA 19104, USA; 2 Department of Biomedical Sciences, University of South Carolina School of Medicine Greenville, Greenville, SC 29605, USA; 3 Department of Obstetrics and Gynecology and the Center for Research on Reproduction and Women’s Health, Perelman School of Medicine, University of Pennsylvania, Philadelphia, Pennsylvania 19104, USA

**Keywords:** embryo, qPCR analysis, blastocoel fluid, fluorescence threshold, variable expression, comparative *Ct*

## Abstract

The use of quantitative PCR (qPCR) and other polymerase chain reaction (PCR)-based methods in the field of human *in vitro* fertilization blastocoel fluid analysis can potentially be utilized for assisting clinicians in embryo selection based on specific gene expression patterns. Since typical Comparative cycle threshold (*C_t_*) analysis utilizes one threshold for runs per gene target and requires an inherent control group, this method is inadequate for analysis of small stochastic systems, such as embryonic-derived fluid. We mathematically demonstrate analytical modifications upon the Comparative *C_t_* qPCR workflow to incorporate a variable fluorescence threshold (utilizing only the parameters defined in the Comparative *C_t_* method), and subsequently demonstrate the typical workflow in which this modified method can successfully quantifiably analyze embryonic blastocoel fluid qPCR analysis.

## Introduction

### Use of qPCR for embryonic blastocoel fluid analysis

For human embryos generated via *invitro* fertilization (IVF), the potential use of an iteration of pre-implantation embryo genetic testing that utilizes non-embryonic components to predict the embryo’s implantation potential or ploidy status represents a developing idea with the potential to increase successful implantation and pregnancy [1]. One clear target for such analysis is embryonic blastocoel fluid, the fluid within the blastocoel cavity of a developing embryo, which (i) is extruded from an embryo during biopsy, (ii) is typically discarded, and (iii) is a proven recognized source of cell-free biological macromolecules such as cell-free DNA, mRNA, and miRNA [2]*.* These characteristics therefore indicate blastocoel fluid as a prime fluid for predictive gene expression analysis, which can be conducted in a noninvasive manner. Currently, there is great interest in identifying molecular indicators within this fluid that would suggest to a reproductive specialist that a given IVF-generated embryo may have a greater chance of leading to a successful pregnancy when compared within a set of the patient’s embryos. In clinical application, this could likely take the form of a rapid qPCR-based assay of blastocoel fluid extruded from embryos, which would theoretically output relative expression within all developed embryos. Genes would be selected based upon ability to predict implantation or genetic status of the embryo based upon rates of expression, due to activity in critical pathways, such as apoptosis. This identification may help reduce the miscarriage rate of IVF patients by offering additional embryonic information *a priori*. While a method to quantify these gene expression levels within embryonic blastocoel fluid is essential, current methodologies (specifically the Comparative cycle threshold (*C_t_*) method) do not have the capacity to quantifiably compare expression in blastocoel fluid samples, for reasons delineated below. Within this article, we delineate a methodology by which we are able to utilize qPCR to study variable levels of gene expression. While we do not claim to advocate the clinical use of this methodology, this method can be utilized to investigate the potential utility – and if confirmed – the clinical application of blastocoel fluid analysis.

### The comparative *C_t_* method

Within the context of living systems, the need to discover relative rates of gene expression is a primary method for determining extent of systemic biological activity. Quantitative PCR (qPCR) and similar qPCR technologies (such as real-time qualitative PCR) have become the flagship methods for gene expression analysis, due to their high sensitivity and versatility [3]. Moreover, qPCR analysis fits the timeframe and efficacy of clinical practice, whereas other methods such as RNAseq are currently more costly and require more time for data collection and analysis than what would be feasibly be expected for a clinically utilizable methodology.

Multiple models of qPCR data analysis have been proposed [4], categorized into “absolute” and “relative” quantification methods. Absolute quantification involves normalization of qPCR data to a determined standard curve. Alternatively, relative quantification methods involve analysis of relative quantities of expression for differing sample groups, typically utilizing both a control gene and a control sample as a comparative standard. The comparative *C_t_* method is a common method for analyzing relative quantification of qPCR studies [5]. This method necessitates two assumptions. First, polymerase chain reaction (PCR) efficiency is universally assumed to be 100%, meaning PCR efficiency remains 2 for every cycle (every strand of DNA exactly doubles per cycle). This is not universally supported, and improvements have been suggested (reviewed by [4, 6, 7] – improvements have also been suggested in other areas such as normalization and kinetics [8, 9]. Second, the relative expression of normalization genes throughout all sample groups (both in the experimental and control groups) is assumed to be constant.

Regardless of method chosen (including both absolute and relative comparison), the fundamental goal persists: to be able to compare the levels of expression between samples. We focus upon the Comparative *C_t_* method in order to describe the most likely utilized method, and which is one that forms the basis for many other methods.

Comparative *C_t_* analysis reports fold change differences in gene expression using a double normalized measurement: The first normalization (intra-sample normalization) occurs between (i) the target gene and (ii) a normalizing (housekeeping) gene (defined as the internal control – intended to be consistently expressed across samples) for all sample groups – thus yielding Δ*C_t_* values. The second normalization (inter-sample normalization) involves comparison of sample (and its associated genes via Δ*C_t_*) against another (typically control) sample (and its associated Δ*C_t_*: defined as the external control) to determine fold change expression difference relative between samples, with fold change expressed as ΔΔ*C_t_* and defined as:

$$\mathrm{Fold}\;\mathrm{Change}=\;\frac{\mathrm{Expression}\;\mathrm{of}\;\mathrm{Gene}\;\mathrm{in}\;\mathrm{Experimental}\;\mathrm{group}}{\mathrm{Expression}\;\mathrm{of}\;\mathrm{Gene}\;\mathrm{in}\;\mathrm{Control}\;\mathrm{Group}}$$

However, the current means of qPCR analysis poses several challenges for blastocoel fluid analysis workflow (Box 1), virtually regardless of method of analysis. While a typical experiment may successfully compare all experimental groups with an inherently stable control group, this is not possible for embryo analysis. What would classify as a control fluid sample? While one may attempt to rationalize categorizations such as “euploid” or “implanted embryo” as control or use aggregated samples as a control group, the blastocoel fluid for these samples is just as arbitrary as any other group. There is only one “control” type system, which is to utilize the difference in expression between different groups which are already confirmed, thus creating a yes/no style of test; however, as expected, this does not fit in any current system of analysis. Moreover, expression of genes may occur at low or high levels, with expression reported in our lab spanning a fluorescence threshold seven-figure ratio. These difficulties make the typical qPCR analysis workflow nearly impossible to successfully replicate in blastocoel fluid studies.

Box 1: Challenges that must be addressed to convert typical qPCR workflow for clinical applications, which prevent direct use of blastocoel fluid studies.

**A lack of a true control group**

There is no expected value of gene expression within embryonic blastocoel fluid. The stochasticity of samples across groups expanded due to the small size of developing embryos and yet again by utilizing cell-free fluid implies that no control can be utilized without having to ignore samples solely upon a stochastics basis.

**Immense variability in samples**

This stochasticity carries over into all target genes by creating variability in expression. One cannot rely upon expression to be present at a certain level, or be totally absent, but rather the expression may be at many points.

**Unexpected amplification or lack thereof**

Some target genes may amplify while others fail to do so. This is an unpredictable reality for qPCR analysis with blastocoel fluid.

**Lack of complete valuation upon one florescence threshold**

Due to the stochasticity of the samples, a single fluorescence threshold does not fit every sample, though all current analysis methods rely upon a constant fluorescence threshold.

### Basis for challenges arising in blastocoel fluid analysis

Generally speaking, within a biological system, intrinsic noise and variability increases with lower numbers of cells. This simple concept clearly demonstrates the issue: a small number of cells in samples results in the system’s inability to suppress stochastic results within a sample. Embryos present the ultimate predictive challenge. Clinicians are presented with (at maximum) hundreds of cells, and expected to base a predictive judgment upon this low number of cells. One cannot simply increase the number of cells in an embryo. The problem is further highlighted for blastocoel fluid, a sample group whose gene expression is highly dependent upon simply the number of lysed cells within an embryo. Thus, creativity is required to develop a fundamental meta-method for qPCR in such a situation. The most challenging aspect of analysis is the fact that the stochasticity in samples can lead to highly variable expression, and without a movable threshold, analysis would likely miss samples.

### Derivation of fluorescent-dependent comparative *C_t_* method

First, we demonstrate a threshold-dependent iteration of Comparative *C_t_* analysis for blastocoel fluid analysis, and do so without introducing any additional restrictions or constraints. Let *N* be the amount of cDNA after a certain number of cycles, where *N_0_* represents the initial cDNA concentration prior to any amplification. Let *F* be fluorescence (as detected by qPCR). Let *E* be efficiency of PCR – assumed to be two for the comparative *C_t_* method as well as this method i.e. after each cycle, it is assumed that the number of DNA exactly doubles per cycle. Fluorescence is defined as:

(1) $$F(t)=\mathrm{kN}(t)=kN_{o}E^{\mathrm{Cyc}(t)}$$

where *k* is an assay-specific gene expansion efficiency constant. At a specific point defined by the fluorescence threshold $F(t)=F_{t}$ at which point cycle value is the cycle threshold *C_t_*, which notably can occur at a fractional number of cycles,

(2) $$F_{t}=kN_{o}E^{C_{t}}$$

where *F_t_* = Fluorescence threshold, *C_t_* = Cycle threshold. For the purpose of method derivation, suppose the calculation is regarding the comparison of expression for a target gene between two samples, and fluorescence and cycle thresholds are provided as would be expected (Table 1).

**Table 1:** This table presents the cycle threshold and fluorescence threshold that one can export from an RT system post-run

Sample	Gene	Cycle threshold	Fluorescence threshold
Embryo 1	Endogenous Control	$C_{t\;\mathrm{Ctrl}S1}$	$F_{t\;\mathrm{Ctrl}S1}$
Embryo 1	Target Gene	$C_{t\;\mathrm{Tar}S1}$	$F_{t\;\mathrm{Tar}S1}$
Embryo 2	Endogenous Control	$C_{t\;\mathrm{Ctrl}S2}$	$F_{t\;\mathrm{Ctrl}S2}$
Embryo 2	Target Gene	$C_{t\;\mathrm{Tar}S2}$	$F_{t\;\mathrm{Tar}S2}$

If *C_t_* is “undetected,” this simply signifies the fact that the sample never reached the threshold value. However, if *C_t_* is detected, values should appear and can thus be compared to other expression values.

With qPCR equations between two samples (each with experimental and control), the four equations then lead to the fold-change ratio (full derivation in Appendix 1, with typical Comparative *C_t_* derivation provided for reference in Appendix 2) of:

(3) $$\frac{\frac{N_{0_{\mathrm{tarS}1}}}{N_{0_{\mathrm{ctrlS}1}}}}{\frac{N_{0_{\mathrm{tarS}2}}}{N_{0_{\mathrm{ctrlS}2}}}}=\frac{F_{t\;\mathrm{tarS}1}}{F_{t\;\mathrm{ctrlS}1}}\frac{F_{t\;\mathrm{ctrlS}2}}{F_{t\;\mathrm{tarS}2}}2^{-\Delta \Delta C_{t}}$$

With $\Delta \Delta C_{t}$ being defined as:

(4) $$\Delta \Delta C_{t}=\Delta C_{t\;S1}-\Delta C_{t\;S2}=\left(C_{t\;\mathrm{tarS}1}-C_{t\;\mathrm{ctrlS}1}\right)-\left(C_{t\;\mathrm{tarS}2}-C_{t\;\mathrm{ctrlS}2}\right)\;$$

This fold-change ratio signifies the normalized proportion of gene expression, as a ratio of target to control between two samples. Note the fact that the consideration of fluorescence threshold simply adds a fluorescence-based coefficient to the front of the typical Comparative *C_t_* equation. Additionally, the method requires no additional assumptions and therefore carries no additional necessary constraint or limiting factor.

This equation symbolizes a formalized means to comparatively understand the impact of variable fluorescence threshold upon a sample group. However, for practical purposes, we derive an even further simplified and utilizable method, the Threshold Consolidation Method.

### Derivation of the threshold consolidation method

Utilizing theoretical qPCR trajectory expansion, one may be able to reconsolidate the thresholds during analysis in another simpler and more pragmatic manner, which is derived by asking what the *C_t_* value would be if the sample expression was “as if” it reached a certain fluorescence threshold. In contrast from the above method, which is a fundamental theoretical means of verifying the possibility of threshold consideration, this method, which is more easily integrated into direct workflow, is intended to be utilized as a transition between data collection and analysis.

Suppose the Fluorescence threshold (*F_t_*) and cycle threshold *C_t_* were determined. The question arises whether one can determine *C_t_*_new_ if this initial *F_t_* and *C_t_* combination were to be mathematically (and not empirically) reassessed at a new threshold *F_t_*_new_, an arbitrary number which one may set equal for all runs, thus accommodating any threshold at which a sample is located For this calculation, the amount N_0_ in both the pre-and post-threshold change is identical so the resulting equality is:

(5) $$\frac{F_{t}}{E^{C_{t}}}=\frac{F_{t\;\mathrm{new}}}{E^{C_{t\;\mathrm{new}}}}$$

And with *E* = 2 (if other efficiencies are determined, this is where they may be implemented in this system),

(6) $$C_{t\;\mathrm{new}}=\log_{2}\left(\frac{F_{t\;\mathrm{new}}}{F_{t}}*2^{C_{t}}\right)\;$$

**Figure 1:**
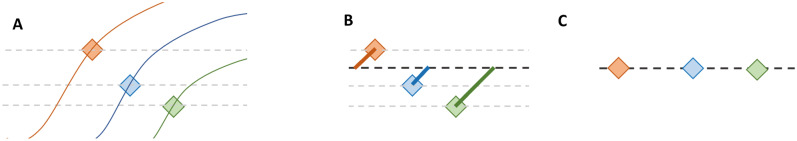
Methodological process behind variable fluorescence calculation. (**A**) During data collection, scientists utilize a portion of qPCR curve which best embodies the general shape of the curve for each analysis per gene – this graphic embodies three samples, each studying one (the same) gene. This does not have to occur at one single threshold. Note that in this workflow, all qPCR curves appear to be smooth, but this is not an accurate assumption for all runs. (**B**) Transition: data points are reevaluated (mathematically) to a single fluorescence threshold (using threshold consolidation method). (**C**) Analysis: comparative *C_t_* analysis occurs on dataset, which now provides *C_t_* values at one threshold (per gene).

This becomes a means by which the variable fluorescence threshold can easily be converted into a common threshold. Figure 1 depicts the change in a visual format. This method carries a nice benefit in that the experimental method has the capability of utilizing a variable threshold, and then subsequently following conversion to a single threshold, the values can then undergo direct Comparative *C_t_* analysis.

## Materials and methods

The focus of this article is not to advocate for a specific qPCR protocol for blastocoel fluid. There are numerous methods to conduct a qPCR method [10,11], and the purpose of this article is to detail a post-analysis analytical (mathematical) method that makes the qPCR data for blastocoel fluid amenable to quantification. Given that this is a generalized post-analysis method meant to cover many forms of nucleic acid expression, including DNA, mRNA, and micro-RNA, this article does not endorse nor provide any specific method for the generation of data for qPCR. To maintain focus upon the innovation itself, we focus upon the data modifications post-data collection.

However, in order to facilitate this discussion, it is critical to maintain a substantive and real example dataset with which to conduct the study. The results of this analysis have been demonstrated with RNA expression from blastocoel fluid. We provide details for out method involved in the collection and presentation of such data below.

### Method

#### Blastocoel fluid collection

Biopsied trophectoderm cells were removed by pipette for preimplantation genetic testing for aneuploidies analysis from intracytoplasmic sperm injection-generated day-5 embryos. Upon completion of the biopsy procedure, the embryo collapses which results in blastocoel fluid being extruded out into the surrounding biopsy medium. Blastocoel-fluid conditioned media (20–25 μl biopsy medium plus blastocoel fluid for each embryo) from IVF-generated embryo culture dish was transferred into a PCR tube, and the media was then cryopreserved at −20 to −80°C until time of analysis, including during storage. An alternative method of blastocoel fluid collection not utilized in this study is collection via blastocentesis.

#### cDNA synthesis

During time of analysis, the thawed blastocoel fluid-conditioned media was subsequently treated with RNase-free DNase 1 for 30 min at 37°C. The DNase was subsequently inactivated by incubation at 65°C for 10 min. Subsequently, cDNA synthesis – formation of DNA via reverse transcription from RNA within the blastocoel fluid – was performed per manufacturer’s instructions (High-Capacity cDNA Reverse Transcription Kit, Applied Biosystems, USA) for each sample. cDNA obtained from each blastocoel fluid sample was diluted in 540 ml of nuclease free water and combined with 540 ml of 2X TaqMan Master Mix (Applied Biosystems, USA). cDNA-Master Mix (10 µl) was then added to each well in the 96-well qPCR plate and prepared for thermal cycler as per manufacturer’s instructions. Each plate was run using a 7500 Fast Real-Time PCR System (Applied Biosystems, USA) at 50°C for 2 min, 95°C for 20 s, followed by 40 cycles of 95°C for 3 s and 60°C for 30 s, all as per manufacturer’s instructions. cDNA from each blastocoel fluid-conditioned media sample was utilized for analysis via qPCR in a 96-well plate using a 7500 Fast Real-Time PCR System. We present results utilizing both gene-specific TaqMan assays and TaqMan Fast Array-Human Apoptosis plate (Applied Biosystems, USA).

Output curve visualizations were analyzed, with a specific focus upon obtaining the *C_t_* of the sample and the threshold at which the sample was well-observed. This involved disabling the *auto-threshold* option, and instead manually adjusting the threshold for all samples. Samples that experienced no expression were also noted.

#### Data analytics

The Threshold Consolidation Equation (Equation (6)) was utilized to determine a new *C_t_* value for all runs at an arbitrary value.

Subsequently, *C_t_* of the normalizing gene was subtracted from the *C_t_* of all target runs:

(7) $$\Delta C_{t}=C_{t\;\mathrm{target}}-C_{t\;\mathrm{control}}$$

The ratio difference in expression between any two samples could then be determined as so

(8) $$\frac{\mathrm{Expression}\;S\mathrm{ample}\;1}{\mathrm{Expression}\;\mathrm{Sample}\;2}=2^{\Delta C_{t}\left(\mathrm{Sample}\;2\right)-\Delta C_{t}(\mathrm{Sample}\;1)}$$

The presence of a 1:1 expression ratio is clinically relevant. However, we also present bulk data with >2 samples worth comparing. There are many means of doing so, including using raw $\Delta C_{t}$ values (Fig. 3B), as well as comparing sample expression fold change ratios (calculated with Equation (8)) all calculated versus one arbitrarily chosen sample (e.g. Sample 1 versus Sample 4, 2 versus 4, 3 versus 4, etc.).

### Ethics approval

Research approval was granted by the Institutional Review Board (IRB) of the University of South Carolina Office of Research Compliance. The study itself is conducted as Not Human Research (since the biopsy fluid samples are normally discarded and is also de-identified) set forth by the Code of Federal Regulations (45 CFR 46) and therefore exempt from further IRB review. The collection of blastocoel-fluid conditioned media was conducted under informed patient consent. The informed consent for treatment (American Society for Reproductive Medicine-Society for Assisted Reproductive Technology (ART) consent template; asrm.org) was modified to include that any unused biological material may be used for current or future research.

## Results

We present three unique results aimed to demonstrate important characteristics of the method. First, we demonstrate the need to utilize a method, which normalizes threshold. Second, we demonstrate what results may theoretically (clinically) be visualized as. Finally, we demonstrate the workflow of the method with a sample data set of blastocoel fluid samples.

### Visualizing the problem

**Figure 2:**
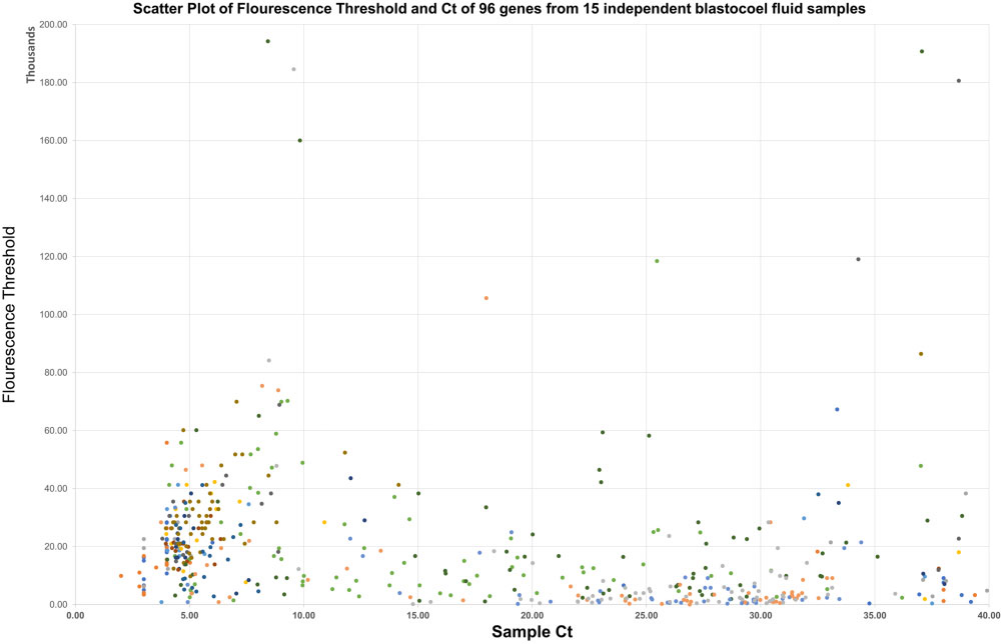
A superposition of gene expression for 16 independent blastocoel fluid samples (with different colors representing different samples) for 94 genes of interest (96-well TaqMan Fast Array-Human Apoptosis plate) is broadly stochastic, with expression covering essentially the entire range of 2–40 for *C_t_* and 0 to 100 000 for fluorescence threshold. Unique colors are associated with unique samples. It would be extremely challenging, if not impossible to systematically account for all points per gene to be located upon a single threshold during analysis – this would inherently eliminate some samples that should not be otherwise.

In order to demonstrate the presence of the problem (namely that there is a near impossibility of coordinating all data onto stationary fluorescence thresholds), we assessed the gene expression *C_t_* value for 90+ apoptosis related genes using a 96-well TaqMan Fast Array-Human Apoptosis plate (Fig. 2) for 16 blastocoel fluid samples.

The figure provides direct evidence to the claims of stochasticity in the gene expression in blastocoel fluid that makes traditional (constant-threshold) analysis impractical, especially since variability clearly only increases with the number of samples analyzed. Our method can help expand and empower analytical methods to go beyond a yes/no-analysis (which is the most ideal outcome of traditional qPCR utilization) into true quantitative analysis. A simple *posthoc* modification would allow for full quantitative analysis of all expressing samples. It is self-evident that the utilization of a variable fluorescence threshold is critical for analysis of stochastic blastocoel fluid to occur, with the levels of expression maintaining such high variability that a single threshold would only be able to account for only a portion of samples at best.

### Sample data

**Figure 3:**
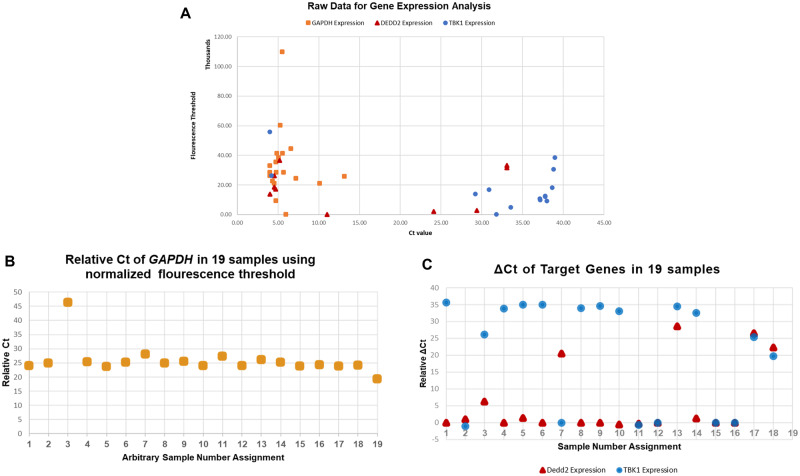
(**A**) Raw data obtained by sample collection for 19 blastocoel fluid samples, which is not normalized or adjusted. (**B**) Normalized housekeeping gene expression after normalization to the same threshold. Note the consistency in expression of housekeeping gene expression, juxtaposed by anticipated variability of target genes. (**C**) Expression of two target genes (*Dedd2* and TBK1) plotted visually as *C_t_* values. The variability in such gene or se of genes is what would form the predictive mechanism behind the method. Note also that despite Sample 3 maintaining a higher *C_t_* than other samples, the relative expression of *Dedd2* and *TBK1* are not both frameshifted upward since normalization accounts for such variability in initial RNA values and serves to correct it. Quantitative values are offered in Supplementary Table S1.

We obtained 19 blastocoel fluid samples from 19 different embryos, and ran qPCR analysis with two genes to demonstrate the utilization of this method (the ploidy status of the embryos, while known, are not reported in this methods paper). The output from qPCR is visualized in Fig. 3A. As expected, we visualize the expected consistency of normalizing/household genes once it is set to a constant fluorescence threshold (Fig. 3B). Conversely, the relative variability of target genes normalized to the housekeeping genes is depicted post-normalization (Fig. 3C). This visualization offers confirmation of general expected consistency in values of fluorescence threshold. While one sample contains a significantly higher *C_t_* than other samples, the fact that this sample’s target gene Δ*C_t_* is within range of other samples provides confirmation in the proper normalization (if unnormalized, the *C_t_* value of the target genes would be shifted upward just as is observed with the housekeeping gene).

#### Sample data handling and analysis

**Figure 4:**
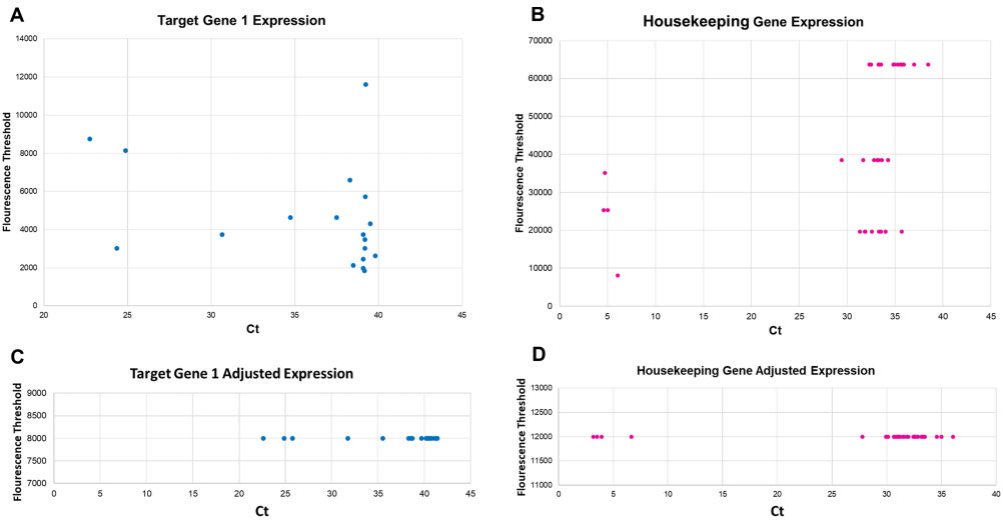
Plot of sample consolidation and visualization for a single gene analysis. (**A**) Plot of *C_t_* value against fluorescence threshold for target gene. (**B**) Plot of *C_t_* value against fluorescence threshold for housekeeping gene. Specific data collection represents our best attempt to consolidate gene expression manually into the smallest number of thresholds, yet this still required multiple thresholds (>3), thus demonstrating the need for a variable threshold. (**C**) A mathematically consolidated sample transformed onto one (arbitrary, but importantly singular) threshold, thus “flattening” the data into one dimension. (**D**) Similar transformation for data for 4B onto one threshold.

Within this subsection, we demonstrate how to utilize data generated from qPCR and adjust it for threshold-modified normalization and subsequent analysis. For this presentation of workflow for certain genes of interest, we presuppose that a certain gene (Target Gene 1) has been detected as importantly correlated with implantation potential, or another clinically relevant facet of ART. Suppose that a clinician must decide between a large number of potentially utilizable oocytes, in this case 36 embryos (which is intentionally large to accommodate all cases, and also to demonstrate the facility of the method with any number of samples), and must determine the relative expression within the group. For this analysis, we utilized a gene indirectly involved in embryonic development via apoptosis-regulation (labeled as Target Gene 1), and utilized a common control gene as a control group (all data provided in Supplementary Table S1). Sample collection occurred manually with the selection of unique thresholds and fluorescence curves that best represented the data. These data are visually represented in Fig. 4A and B, with *C_t_* plotted against fluorescence threshold. Note that as expected, the stochasticity of the target gene is higher than that of the control gene.

Step 1: Consolidate data to one fluorescence threshold: Without the ΔΔ*C_t_* method, all gene expression would have to occur in one horizontal line (across one single fluorescence threshold). However, it has been claimed in this article that prior pure Comparative *C_t_* method-based analysis will forfeit samples that will not be able to be plotted upon that threshold. This can be demonstrated by understanding the morphology of data points of Fig. 4B. During the collection of data for this figure, we attempted our best to ensure that all data points were collected from a similar threshold. Unfortunately, the best we could do was to utilize only three drastically different thresholds (19 668, 38 452, and 63 659) and collect *C_t_*-values on those points. Since these samples characteristically demonstrate late-*C_t_* expression, this figure visually proves the claim that a single fluorescence threshold is incapable of accounting for all samples. This does not imply that our housekeeping gene is stochastic, but rather that the biological system is. Suppose that the concentration of DNA depends upon the level of apoptosis in the blastocoel fluid. In such an instance, which may be a major contributing factor for expression, the number of cells undergoing apoptosis may directly influence the concentration of housekeeping gene, even if the expression level of housekeeping genes is constant.

However, we demonstrate here how we can utilize a variable fluorescence threshold and convert the samples into a 2D-linear array that can then be utilized for subsequent analysis. One can easily utilize Equation (6) to collapse samples onto one threshold as is done for target genes (from Fig. 4A–C) and control genes (Fig. 4B–D). Adjusted fluorescence thresholds have been arbitrarily chosen as 8000 and 12 000, though it is notable to claim that the individual fluorescence chosen is irrelevant for the final calculation so long as it consistent for all individual points per gene.

**Figure 5:**
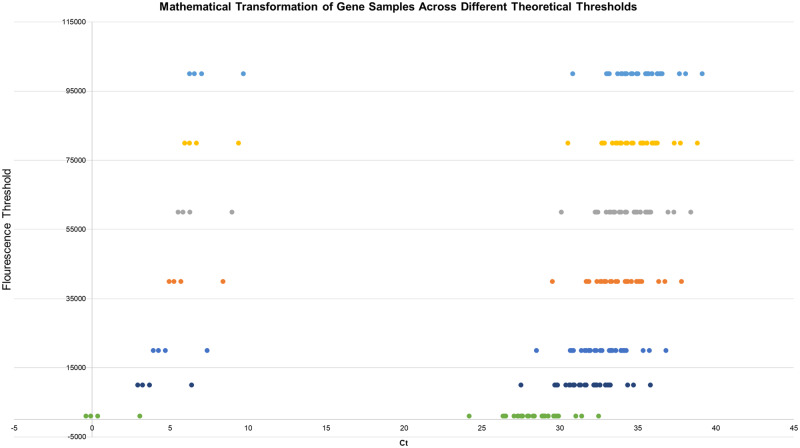
Demonstration of threshold variability for control genes across varying thresholds (specifically 1, 10, 20, 40 60, 80, and 100 thousand). While the logarithmic transformation creates a unique pattern in transformation, the critical aspect is the fact that the relevant difference in samples is unchanged regardless of threshold, thus demonstrating the arbitrariness of the specific threshold used for analysis.

In addition, with our dataset, we can visually demonstrate the fundamental disutility of any specific fluorescence threshold. We can plot the same data from Fig. 4B onto multiple fluorescence thresholds (Fig. 5), and demonstrate that the fundamental *change* in *C_t_* is equal regardless of specific fluorescence threshold.

Step 2: Normalize *C_t_* values within samples, then compare against other samples. The first of these procedures simply involves subtracting the value of the *C_t_* for the housekeeping gene from the target gene, which forms the Δ*C_t_* via Equation (7). These values can subsequently be compared to one another by multiple means, one of which we describe below. We develop a fold change ratio by using Equation (8), and specifically comparing the expression of all samples to the expression of one arbitrarily chosen sample (we used Sample 4 since it presented the highest expression, though it is important to note that the relative comparative ratios would remain identical had we chosen any other sample to compare with).

Whereas regular analysis would only be able to tell us which embryos had expression and which lacked expression (in a yes/no manner similar to Supplementary Table S2), analysis of genes with expression with our method allows for quantitative relative rates of expression (Table 2).

**Table 2:** Relative rates of expression (in the second column), followed by the logarithmic value of the fold change ratio (third column), which is simply calculated by taking the logarithm of the fold change

Sample	Fold change ratio	Log(fold change)
Expressed Sample 1	7.06E-06	−5.15
Expressed Sample 2	0.16	−0.80
Expressed Sample 3	1.70E-04	−3.75
Expressed Sample 4	1.00	0.00
Expressed Sample 5	6.51E-06	−5.19
Expressed Sample 6	1.40E-03	−2.85
Expressed Sample 7	9.76E-07	−6.01
Expressed Sample 8	0.087	−1.06
Expressed Sample 9	1.88E-13	−12.73
Expressed Sample 10	1.36E-05	−4.87
Expressed Sample 11	1.22E-06	−5.91
Expressed Sample 12	1.41E-06	−5.85
Expressed Sample 13	1.42E-06	−5.85
Expressed Sample 14	1.07E-14	−13.97
Expressed Sample 15	3.12E-07	−6.51
Expressed Sample 16	5.18E-05	−4.29
Expressed Sample 17	1.14E-06	−5.94
Expressed Sample 18	1.01E-06	−5.99

**Figure 6:**
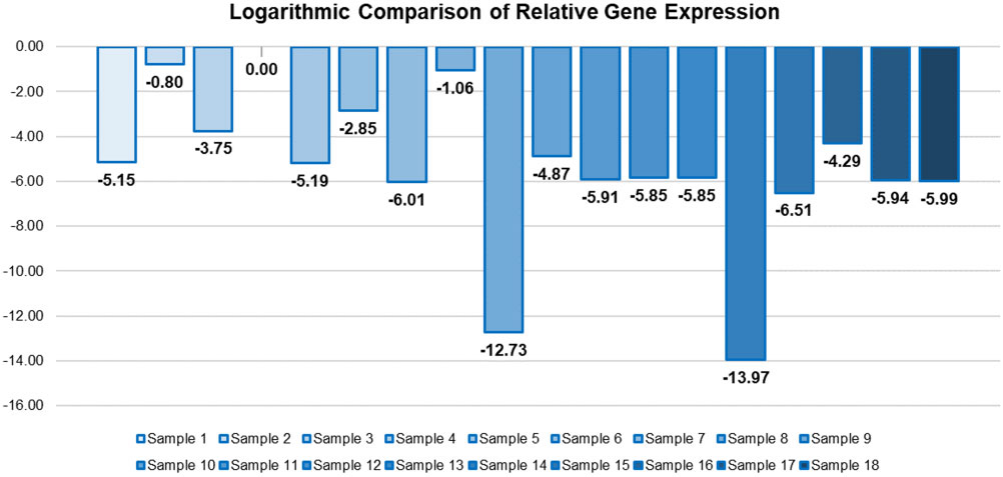
Logarithmic demonstration of relative expression between multiple samples.

Utilizing the logarithmic values in Table 2, we can plot the logarithmic relationship of expression within samples that present any form of expression (Fig. 6). This reveals what we hoped to see, a clear and quantitative demarcation of relative expression between all samples.

This is the quantitative outcome that is impossible to attain without a variable threshold – recall that these results were derived from normalization from a housekeeping gene, and with both the housekeeping and target genes being able to vary across different thresholds as other samples. The removal of the constant fluorescence threshold, a seemingly arbitrary relic of the Comparative *C_t_* method is essential in enabling the comparison of expression rates that are so fundamentally different that they cannot be reconciled into one threshold, for example with Sample 4 (highest expressing) and Sample 14 (lowest expressing) from Fig. 4 demonstrating a ∼10^14^ level difference in expression compared to the highest expressing sample 4. Notably, even the lowest-expressing sample is expressing some of the gene, unlike other samples, which simply failed to express at all. Such nuanced distinctions are critical for qPCR to be clinically utilizable, and this implies the necessity of a variable threshold.

## Discussion

We present the fluorescence dependent Comparative *C_t_* method, which offers a pathway of analysis of assessing gene expression in blastocoel fluid-conditioned media. Development of a means to assess the differences in molecular components of blastocoel fluid derived from IVF-generated embryos of varying ploidy or implantation status is of great clinical interest. The ability to detect a specific gene expression pattern in blastocoel fluid may provide embryologists with another piece of molecular information to use when selecting the most viable IVF-embryo for transfer. This article provides a method for accurately analyzing this type of gene expression data collected with qPCR.

This novel method of analysis is able to fully address every challenge that a clinical utilization of qPCR raises:

1. Immense variability and variable fluorescence threshold: Our threshold consolidation achieves the effect of nullifying any issues with even the most variable thresholds;
2. Inherent control group: We implicity resolve this issue, since the use of all prior samples as a comparative group serves as a viable means of validation in blastocoel fluid studies. There is no “normal” or “expected” concentration of any target gene in blastocoel fluid. Our method determines means to achieve this result; and
3. Lack of amplification: We address this issue with the explicit inclusion of samples that do not have any amplification into our protocol.

While the applications of qPCR may be the most sensitive way in which expression information regarding gene expression informatics may be derived, the technique and method of analysis must undergo modification to be clinically utilized in embryo utilization prediction. At this time when a non-invasive means of assessing IVF-embryo viability is being intensely investigated, this distinct analysis protocol will allow for gene expression to be included in this pursuit.

This article does not advocate for the clinical implementation of this methodology or for blastocoel fluid analysis. Doing so would require both more data as well as demonstration of reproducibility, which we have not shown in this article. Instead, we have demonstrated the means by which this analysis may take place by qPCR.

One potential area for growth of this method involves the level of work required for data collection. Unfortunately many systems currently lack a means of quickly exporting threshold values with *C_t_*, and as such this must be done manually. However, this purely technical issue is one that may easily be resolved. Perhaps a greater challenge is the fact that this method relies upon ideal 2-fold amplification in every cycle. Yet, this is not a challenge that the proposed method in this article raises, but one that affects all Comparative *C_t_* methods. The mathematical adjustment was made with the exact same conditions and limitations offered in the typical Comparative *C_t_* method, and so it is unsurprising that recent adjustments to variables such as efficiency can easily carry over into this method as well.

The closed-form utility of the math behind this method implies that the Fluorescence-Dependent Comparative *C_t_* method can – without modification – be expanded to other fields in molecular and cellular biology which utilize qPCR, including in confirmation of RNAseq analyses, among others. Moreover, as samples being utilized become smaller in volume, potentially even down to the single cell or cell-free fluid, the likelihood for stochastic variability increases, and thus a variable threshold may be able to accommodate and account for changes in gene expression that traditional methods may not be able to quantify.

## Supplementary Material

bpab001_Supplementary_Data

## Data Availability

Data for Figs 3 and 5 are provided in supplementary table.

## Appendix 1

Two experimental analyses with target and control runs yield four equations:

$$C_{t\;\mathrm{ctrl}S1}=\log_{2}\left(\frac{F_{t\;\mathrm{ctrl}S1}}{k_{\mathrm{ctrl}}}\right)-\log_{2}\left(N_{0_{\mathrm{ctrl}S1}}\right)\;C_{t\;\mathrm{ctrl}S2}=\log_{2}\left(\frac{F_{t\;\mathrm{ctrl}S2}}{k_{\mathrm{ctrl}}}\right)-\log_{2}\left(N_{0_{\mathrm{ctrlS}2}}\right)$$

$$C_{t\;\mathrm{tarS}1}=\log_{2}\left(\frac{F_{t\;\mathrm{tarS}1}}{k_{\mathrm{tar}}}\right)-\log_{2}\left(N_{0_{\mathrm{tarS}1}}\right)\;C_{t\;\mathrm{tarS}2}=\log_{2}\left(\frac{F_{t\;\mathrm{tarS}2}}{k_{\mathrm{tar}}}\right)-\log_{2}\left(N_{0_{\mathrm{tarS}2}}\right)$$

We first normalize target with endogenous control gene for both samples.

$$C_{t\;\mathrm{ctrlS}1}-C_{t\;\mathrm{tarS}1}=\log_{2}\left(\frac{F_{t\;\mathrm{ctrlS}1}}{k_{\mathrm{ctrl}}}\right)-\log_{2}\left(N_{0_{\mathrm{ctrlS}1}}\right)-\log_{2}\left(\frac{F_{t\;\mathrm{tarS}1}}{k_{\mathrm{tar}}}\right)+\log_{2}\left(N_{0_{\mathrm{tarS}1}}\right)$$

$$C_{t\;\mathrm{ctrlS}1}-C_{t\;\mathrm{tarS}1}=\log_{2}\left(\frac{F_{t\;\mathrm{ctrlS}1}}{k_{\mathrm{ctrl}}\;}\frac{k_{\mathrm{tar}}}{F_{t\;\mathrm{tarS}1}}\frac{N_{0_{\mathrm{tarS}1}}}{N_{0_{\mathrm{ctrlS}1}}}\right)$$

Similarly,

$$C_{t\;\mathrm{ctrlS}2}-C_{t\;\mathrm{tarS}2}=\log_{2}\left(\frac{F_{t\;\mathrm{ctrlS}2}}{k_{\mathrm{ctrl}}\;}\frac{k_{\mathrm{tar}}}{F_{t\;\mathrm{tarS}2}}\frac{N_{0_{\mathrm{tarS}2}}}{N_{0_{\mathrm{ctrlS}2}}}\right)$$

Defining $C_{t\;\mathrm{tarS}1}-C_{t\;\mathrm{ctrlS}1}$ as $\Delta C_{t\;S1}$ and $C_{t\;\mathrm{tarS}2}-C_{t\;\mathrm{ctrlS}2}$ as $\Delta C_{t\;S2}$,

$-\;\Delta C_{t\;S1}=\log_{2}\left(\frac{F_{t\;\mathrm{ctrlS}1}}{k_{\mathrm{ctrl}}\;}\frac{k_{\mathrm{tar}}}{F_{t\;\mathrm{tarS}1}}\frac{N_{0_{\mathrm{tarS}1}}}{N_{0_{\mathrm{ctrlS}1}}}\right)$ and $-\Delta C_{t\;S2}=\log_{2}\left(\frac{F_{t\;\mathrm{ctrlS}2}}{k_{\mathrm{ctrl}}\;}\frac{k_{\mathrm{tar}}}{F_{t\;\mathrm{tarS}2}}\frac{N_{0_{\mathrm{tarS}2}}}{N_{0_{\mathrm{ctrlS}2}}}\right)$

Converting to exponential terms:

$2^{-\;\Delta C_{t\;S1}}=\frac{F_{t\;\mathrm{ctrlS}1}}{k_{\mathrm{ctrl}}\;}\frac{k_{\mathrm{tar}}}{F_{t\;\mathrm{tarS}1}}\frac{N_{0_{\mathrm{tarS}1}}}{N_{0_{\mathrm{ctrlS}1}}}$ and $2^{-\;\Delta C_{t\;S2}}=\frac{F_{t\;\mathrm{ctrlS}2}}{k_{\mathrm{ctrl}}\;}\frac{k_{\mathrm{tar}}}{F_{t\;\mathrm{tarS}2}}\frac{N_{0_{\mathrm{tarS}2}}}{N_{0_{\mathrm{ctrlS}2}}}$

Defining (identically to the comparative *C_t_* method) $\Delta \Delta C_{t}=\Delta C_{t\;S1}-\Delta C_{t\;S2}$

$$\frac{2^{-\;\Delta C_{t\;S1}}}{2^{-\;\Delta C_{t\;S2}}}=2^{-(\Delta C_{t\;S1}-\Delta C_{t\;S2})}=2^{-\Delta \Delta C_{t}}=\frac{\frac{F_{t\;\mathrm{ctrlS}1}}{k_{\mathrm{ctrl}}\;}\frac{k_{\mathrm{tar}}}{F_{t\;\mathrm{tarS}1}}\frac{N_{0_{\mathrm{tarS}1}}}{N_{0_{\mathrm{ctrlS}1}}}}{\frac{F_{t\;\mathrm{ctrlS}2}}{k_{\mathrm{ctrl}}\;}\frac{k_{\mathrm{tar}}}{F_{t\;\mathrm{tarS}2}}\frac{N_{0_{\mathrm{tarS}2}}}{N_{0_{\mathrm{ctrlS}2}}}}$$

This step has the intended effect of eliminating all constant variables.

$$2^{-\Delta \Delta C_{t}}=\frac{\frac{F_{t\;\mathrm{ctrlS}1}}{F_{t\;\mathrm{tarS}1}}\frac{N_{0_{\mathrm{tarS}1}}}{N_{0_{\mathrm{ctrlS}1}}}}{\frac{F_{t\;\mathrm{ctrlS}2}}{F_{t\;\mathrm{tarS}2}}\frac{N_{0_{\mathrm{tarS}2}}}{N_{0_{\mathrm{ctrlS}2}}}}\;\mathrm{Fold}\;\mathrm{Change}\;\mathrm{Ratio}:\;\frac{\frac{N_{0_{\mathrm{tarS}1}}}{N_{0_{\mathrm{ctrlS}1}}}}{\frac{N_{0_{\mathrm{tarS}2}}}{N_{0_{\mathrm{ctrlS}2}}}}=\frac{F_{t\;\mathrm{tarS}1}}{F_{t\;\mathrm{ctrlS}1}}\frac{F_{t\;\mathrm{ctrlS}2}}{F_{t\;\mathrm{tarS}2}}2^{-\Delta \Delta C_{t}}$$

## Appendix 2

Calculating ΔΔ*C_t_* via Comparative *C_t_* method:

$$Ct=\log_{2}\left(\frac{F_{t}}{k}\right)-\log_{2}\left(N_{o}\right)$$

$${Ct}_{\mathrm{refexp}}=\log_{2}\left(\frac{F_{\mathrm{texp}1}}{k_{\mathrm{ref}}}\right)-\log_{2}\left(N_{0_{\mathrm{refexp}}}\right)$$

$${\mathrm{Ct}}_{\mathrm{tarexp}}=\log_{2}\left(\frac{F_{\mathrm{texp}2}}{k_{\mathrm{tar}}}\right)-\log_{2}\left(N_{0_{\mathrm{tarexp}}}\right)$$

$${Ct}_{\mathrm{refctrl}}=\log_{2}\left(\frac{F_{\mathrm{tctrl}3}}{k_{\mathrm{ref}}}\right)-\log_{2}\left(N_{0_{\mathrm{refctrl}}}\right)$$

$${Ct}_{\mathrm{tarctrl}}=\log_{2}\left(\frac{F_{\mathrm{tctrl}4}}{k_{\mathrm{tar}}}\right)-\log_{2}\left(N_{0_{\mathrm{tarctrl}}}\right)$$

$$\Delta {\mathrm{Ct}}_{\exp}={\mathrm{Ct}}_{\mathrm{tarexp}}-{\mathrm{Ct}}_{\mathrm{refexp}}=\log_{2}\left[\left(\frac{F_{\mathrm{texp}1}}{k_{\mathrm{tar}}}\right)\left(\frac{k_{\mathrm{ref}}}{F_{\mathrm{texp}2}}\right)\left(\frac{N_{0_{\mathrm{refexp}}}}{N_{0_{\mathrm{tarexp}}}}\right)\right]$$

$$\left(\frac{N_{0_{\mathrm{tarexp}}}}{N_{0_{\mathrm{refexp}}}}\right)=\frac{k_{\mathrm{ref}}}{k_{\mathrm{tar}}}\left(\frac{F_{\mathrm{texp}1}}{F_{\mathrm{texp}2}}\right)2^{-\Delta {Ct}_{\;\text{exp}\;}}$$

$$\frac{N_{0_{\mathrm{refctrl}}}}{N_{0_{\mathrm{refexp}}}}=\left(\frac{F_{\mathrm{texp}1}}{F_{\mathrm{tctrl}3}}\right)2^{{\mathrm{Ct}}_{\mathrm{refexp}}-{\mathrm{Ct}}_{\mathrm{refctrl}}}$$

$$\Delta {\mathrm{Ct}}_{\mathrm{ctrl}}={\mathrm{Ct}}_{\mathrm{tarctrl}}-{\mathrm{Ct}}_{\mathrm{refctrl}}=\log_{2}\left[\left(\frac{F_{\mathrm{tctrl}3}}{k_{\mathrm{tar}}}\right)\left(\frac{k_{\mathrm{ref}}}{F_{\mathrm{tctrl}4}}\right)\left(\frac{N_{0_{\mathrm{refctrl}}}}{N_{0_{\mathrm{tarctrl}}}}\right)\right]$$

$$\left(\frac{N_{0_{\mathrm{tarctrl}}}}{N_{0_{\mathrm{refctrl}}}}\right)=\frac{k_{\mathrm{ref}}}{k_{\mathrm{tar}}}\left(\frac{F_{\mathrm{tctrl}3}}{F_{\mathrm{tctrl}4}}\right)2^{-\Delta {\mathrm{Ct}}_{\mathrm{ctrl}}}$$

Setting $\Delta \Delta Ct={\Delta \mathrm{Ct}}_{\exp}-{\Delta \mathrm{Ct}}_{\mathrm{ctrl}}$,

$$\mathrm{Ratio}/\mathrm{Fold}\;\mathrm{Change}:\frac{\left(\frac{N_{0_{\mathrm{tarexp}}}}{N_{0_{\mathrm{refexp}}}}\right)}{\left(\frac{N_{0_{\mathrm{tarctrl}}}}{N_{0_{\mathrm{refctrl}}}}\right)}=2^{-\Delta {\mathrm{Ct}}_{\exp}+\Delta {\mathrm{Ct}}_{\mathrm{ctrl}}}=2^{-\Delta \Delta Ct}$$
