# Supplementary material for: Fluorescent-dependent comparative Ct method for qPCR gene expression analysis in IVF clinical pre-implantation embryonic testing
Source: Biol Methods Protoc. 2021 Jan 18;6(1):bpab001. doi: 10.1093/biomethods/bpab001 (PMC7998709; doi:10.1093/biomethods/bpab001)
Supplement: bpab001_Supplementary_Data [file bpab001_supplementary_data.docx]

**Supplementary Table 1.** Table with C_t_ and ΔC_t_ values for samples utilized in Figure 3. This table contains both raw data and adjusted data.

**Supplementary Table 2.** Table with raw expression values utilized in the creation of Figures 3 and 4. If no fluorescence adjusting occurred, this visual yes/no expression is the greatest level of analytical power that qPCR would provide.
